# One way or another: The opportunities and pitfalls of self‐referral and consecutive sampling as recruitment strategies for psycho‐oncology intervention trials

**DOI:** 10.1002/pon.4780

**Published:** 2018-06-29

**Authors:** Belinda Thewes, Judith A.C. Rietjens, Sanne W. van den Berg, Félix R. Compen, Harriet Abrahams, Hanneke Poort, Marieke van de Wal, Melanie P.J. Schellekens, Marlies E.W.J. Peters, Anne E.M. Speckens, Hans Knoop, Judith B. Prins

**Affiliations:** ^1^ Radboud Institute of Health Sciences, Department of Medical Psychology Radboud University Medical Center Nijmegen The Netherlands; ^2^ Department of Public Health Erasmus MC Rotterdam Rotterdam The Netherlands; ^3^ Karify Utrecht The Netherlands; ^4^ Department of Psychiatry Radboud University Medical Center Nijmegen The Netherlands; ^5^ Academic Medical Center (AMC), University of Amsterdam, Department of Medical Psychology Amsterdam Public Health Research Institute Amsterdam The Netherlands; ^6^ Department of Psychosocial Oncology and Palliative Care Dana‐Farber Cancer Institute Boston MA USA; ^7^ Department of Medical Psychology Máxima Medical Center Eindhoven/Veldhoven The Netherlands; ^8^ Department of Medical Oncology Radboud University Medical Center Nijmegen The Netherlands

**Keywords:** cancer, oncology, psychological interventions, self‐referral, recruitment, eHealth, consecutive sampling

**Key points**

- Consecutive recruitment is an important recruitment strategy in psycho‐oncology interventions trials. However, greater pragmatism is needed.
- Psycho‐oncology interventions differ from many other cancer treatments in that not all cancer patients will want or need a treatment despite experiencing psychological symptoms.
- Self‐referral recruitment might enhance patient‐centred care and help overcome the translational gap in moving evidence‐based interventions from research to reality.
- Self‐referral recruitment can be less resource intensive than clinic‐based consecutive recruitment and may facilitate more rapid attainment of recruitment targets.
- Further debate is needed concerning the ethical aspects of self‐referral recruitment methods

## 1 INTRODUCTION

Recent decades have seen growth in evidence‐based psycho‐oncology interventions (POIs). However, many patients do not receive best‐practice psychosocial care due to a lack of implementation in routine care. Failure to implement may, in part, be because randomised controlled trials (RCTs) study efficacy under highly controlled (“ideal”) conditions. Pragmatism within RCTs can occur along various dimensions (eg, recruitment and delivery), allowing some aspects of RCTs to be more explanatory and others more pragmatic.

This manuscript compares consecutive sampling and self‐referral recruitment methods for POI RCTs, which we define as interventions to manage the psychological, behavioural, and/or social aspects of cancer to promote health. We believe the current preference for consecutive sampling in POI RCTs negatively impacts recruitment and may hamper implementation. Views are based on our recent experience with developing, testing, and implementing POIs.

## 2 CONSECUTIVE RECRUITMENT

Consecutive sampling is considered the best of the nonprobability sampling methods at controlling sampling bias because it includes all available subjects.^1^ In our experience, RCTs using consecutive sampling methods are often favoured by funding bodies and journal editors. In clinical settings, consecutive sampling provides insight into the number of eligible patients (allowing the calculation of a response rate) and enables the use of clinical information. It also provides insight into numbers of patients that might be willing to use a POI, allows calculation of an accurate response rate, and provides an opportunity for health professional endorsement of an intervention that may promote credibility. Although consecutive sampling is widely used in RCTs, it is not mandated in CONSORT guidelines. Despite the importance of recruitment to generalisability, the CONSORT statement does not specify an optimal method of recruitment.

The presence of psychosocial symptoms does not equate with an interest in POI. Therefore, the implicit assumption within consecutive sampling that all patients might want, need, or benefit from an intervention is not valid for POIs. Recent reviews by Wakefield et al^2^ and Brebach et al^3^ report average uptake rates of 60%‐66% in POI RCTs among distressed patients. However, considerably lower rates have been reported in many RCTs. In this context, consecutive sampling can be costly and resource intensive. Van Scheppingen et al^4^ found that of 1038 cancer patients consecutively invited to a POI RCT, only 36 (4% of screened patients) were ultimately randomised requiring 17 hours of nurse/researcher time to recruit one patient.

Psycho‐oncology researchers frequently rely on clinicians to invite patients to POIs, meaning that true consecutive sampling is rarely achieved. Reasons for “gatekeeping” include clinicians forgetting to approach patients, a greater focus on medical problems, a lack of awareness of the potential benefits of POIs, lack of clinician engagement, or fear that research participation will threaten wellbeing. Yet, for instance, over 90% of patients receiving palliative cancer treatment wanted to be informed about fatigue intervention studies.^5^ Consecutive sampling also is not immune to sampling bias, as bias may occur due to commonalities between patients drawn from particular clinics. Given the enormous cost of RCTs and increasing need to consider implementation, consecutive sampling may not always be necessary or even desirable.

## 3 SELF‐REFERRAL RECRUITMENT

Researchers are increasingly considering self‐referral recruitment in POI RCTs. A major advantage of self‐referral is that it provides information about demand and characteristics of patients motivated to participate. With relatively low resource investment, researchers can quickly boost the number of patients recruited. In a climate where recruitment is highly challenging and many POI fail to be implemented in real life, this is a major advantage.

Self‐referral methods might also promote greater self‐management and empowerment. If self‐referred patients are encouraged to discuss research participation with clinicians, it may help educate clinicians about their unmet needs.

Self‐referral might also be particularly useful for overcoming the translational gap from research to reality. Our BREATH RCT of a low‐intensity online CBT‐based self‐management intervention for breast cancer survivors used clinic‐based consecutive sampling.^6^ RCT participants were a representative clinic sample with 68% reporting *low‐medium* distress. The intervention proved beneficial and had greatest benefit in patients with low distress. However, in implementation when access was made available via self‐referral at a public website, 100% of users had *high* distress despite the website advising highly distressed women to contact their GP for more intensive treatment. Due to this discrepancy, positive study results cannot be generalised to actual users of BREATH in routine care. Choosing a recruitment strategy that fits the context of future implementation is therefore crucial to improving ecological validity.

Critics of self‐referral argue that it attracts different patients to those who would be referred by clinicians and the “worried well.” In a systematic review of studies with recruitment through Facebook, 24 of 36 studies compared their sample with population data for representativeness.^7^ Most samples were broadly representative, although more Caucasian, highly educated, younger, females were found in some samples.^7^ This problem is however also common to studies using consecutive recruitment.

Two of our POI using self‐referral found that self‐referred patients are quite similar to those recruited via other methods, differing only in that self‐referred patients included more breast cancer patients^8^ and those with a higher stage of disease.^9^ It is therefore recommended that studies using both self‐referral and consecutive sampling methods compare patient characteristics of patients recruited via each method and be adequately powered to allow subgroup analysis if appropriate.

Disadvantages of self‐referral recruitment are that while it could boost recruitment, lower engagement and higher attrition may be a problem. However, in our RCTs, this has not been the case. In the CHANGE study,^9^ where both consecutive sampling and self‐referral were used, self‐referred patients were not more likely to drop out than clinic recruited patients. More research is needed to explore the impact of recruitment method on attrition and engagement.

While self‐referral can be a feasible recruitment strategy for POI RCTs, it may raise ethical questions with respect to privacy and information sharing. Where possible, researchers should gain patient consent to inform the treating physician of participation and verify eligibility. Self‐referral complicates calculating response rates, hence limiting insight into nonresponse. Social media is an increasingly used and successful recruitment strategy. In addition to the already described advantages, a key advantage is improved participation among groups that can be hard to reach with traditional approaches (eg, younger people and ethnic minorities). However, the ethical aspects of recruitment via social media require careful consideration. Further debate is needed to develop effective, ethical ways to disseminate the information about the availability of POI RCTs and support communication between the patient, researcher, and clinicians. There is also a need to ensure that social and mobile media sampling methods are used in an active but non‐invasive manner.

## 4 WHEN IS SELF‐REFERRAL INDICATED?

Self‐referral might be more suitable for particular types of POIs. For example, self‐management and eHealth POIs usually require relatively high levels of self‐motivation. POI RCTs targeting problems that are often neglected during routine clinical care (eg, sexuality, fatigue, and fear of cancer recurrence) may also benefit from self‐referral recruitment.

Gatekeeping, sampling bias, incomplete data, and attrition are common problems in advanced cancer research. Self‐referral might help address some of these barriers to inclusion. Furthermore, when care increasingly focuses on comfort rather than on cure, patients may visit clinics less frequently, making it harder to reach them through consecutive clinic sampling. As treatment improves, cancer survivors will likely become more similar to the general population in terms of their geographic mobility, potentially limiting cancer registries and clinics as a means of recruitment. Self‐referral may therefore become a better recruitment strategy.

Some types of POI RCTs might be less suitable for self‐referral sampling recruitment (eg, research on patient‐clinician communication interventions) where demonstration of efficacy depends on a broadly representative sample. Due to the potential of sampling bias and the inclusion of patients with a greater level of need for help with psychological problems, self‐referral recruitment might inflate the efficacy of POIs designed for all patients. Furthermore, some patient subgroups might be omitted in RCTs using self‐referral sampling alone, due to poor health literacy, lack of awareness of availability and benefits of treatment, avoidant coping, or perceived stigma accessing psychological treatment. Our recent RCT of mindfulness‐based stress reduction found lung cancer patients valued the chance to discuss participation with their doctor and get support with the decision‐making process about participation.^10^

## 5 IS SELF‐REFERRAL FEASIBLE IN POI RCTS?

Our experience of using self‐referral recruitment in 2 RCTs^8^, ^9^ has resulted in relatively short inclusion periods, attainment of target recruitment, and high participation rates relative to our other RCTs not using self‐referral as a recruitment strategy^6^, ^10^, ^11^, ^12^ (see Table 1). This provides preliminary support for the feasibility of self‐referral in POI RCTs.

**Table 1:** Recruitment experiences in selected recent RCTs

Authors	Name of RCT	Cancer Type (Stage)	Period of Inclusion (Months)	Number of Intended Patients	Number (%) of Recruitment Target Attained	Participation Rate^a^^,^ b	Method of Recruitment	Number (%) of Sample Included Participants Via Self‐Referred
Compen et al^8^	BeMind	Mixed cancer	20	245	245 (100%)	60% (245/410)^a^	Self‐referral and consecutive	181/245 (74%)
Abrahams et al^9^	CHANGE	Breast cancer (stages I‐III)	27	132	132 (100%)	45% (132/291)^a^	Self‐referral and consecutive	56/132 (42%)
van den Berg et al^6^	BREATH	Breast cancer (stages I‐III)	20	170	151 (89%)	89% (151/170)^b^	Consecutive	Not applicable
van de Wal et al^11^	SWORD	Mixed cancer (breast, colorectal, and prostate) Stage data not available.	24	104	88 (85%)	12% (88/750)^a^	Consecutive	Not applicable
Schellekens et al^10^	MILON	Lung cancer (stages I‐IV)	39	110	63 (57%)	18% (63/359)^a^	Consecutive	Not applicable
Poort et al^12^	TIRED	Advanced cancer, mixed cancer (stage IV)	57	161	134 (83%)	58% (134/232)^b^	Consecutive	Not applicable

Abbreviation: RCT, randomised controlled trial.

a Denominator includes all screened and potentially eligible patients.

b Denominator includes only patients screened, eligible patients, and referred by health professional to the study (ie, an unknown number of patients were not screened or not invited or were not interested at the initial invitation.

## 6 CONCLUSIONS

Consecutive recruitment remains an important recruitment strategy. However, greater pragmatism is needed in recruitment to POI RCTs. When designing an RCT, it is essential to consider its intended implementation strategy. Where access to the intervention will be made available via self‐referral in implementation, self‐referral should be considered as a recruitment method. Studies using both self‐referral and consecutive sampling should compare characteristics of patients recruited via each method. Greater use of self‐referral recruitment methods might enhance the provision of patient‐centred care, increase ecological validity, facilitate greater equity of access to POI research, and facilitate faster implementation of effective POIs into clinical practice. However, more debate is needed concerning the ethical aspects of self‐referral recruitment.
